# Do Food Web Models Reproduce the Structure of Mutualistic Networks?

**DOI:** 10.1371/journal.pone.0027280

**Published:** 2011-11-02

**Authors:** Mathias M. Pires, Paulo I. Prado, Paulo R. Guimarães

**Affiliations:** 1 Programa de Pós-graduação em Ecologia, Instituto de Biociências, Universidade de São Paulo, São Paulo, Brazil; 2 Departamento de Ecologia, Instituto de Biociências, Universidade de São Paulo, São Paulo, Brazil; Institut Mediterrani dEstudis Avançats (CSIC/UIB), Spain

## Abstract

**Background:**

Simple models inspired by processes shaping consumer-resource interactions have helped to establish the primary processes underlying the organization of food webs, networks of trophic interactions among species. Because other ecological interactions such as mutualisms between plants and their pollinators and seed dispersers are inherently based in consumer-resource relationships we hypothesize that processes shaping food webs should organize mutualistic relationships as well.

**Methodology/Principal Findings:**

We used a likelihood-based model selection approach to compare the performance of food web models and that of a model designed for mutualisms, in reproducing the structure of networks depicting mutualistic relationships. Our results show that these food web models are able to reproduce the structure of most of the mutualistic networks and even the simplest among the food web models, the cascade model, often reproduce overall structural properties of real mutualistic networks.

**Conclusions/Significance:**

Based on our results we hypothesize that processes leading to feeding hierarchy, which is a characteristic shared by all food web models, might be a fundamental aspect in the assembly of mutualisms. These findings suggest that similar underlying ecological processes might be important in organizing different types of interactions.

## Introduction

A major challenge in ecology is to understand how ecological networks are assembled. Network assembly ultimately reflects how interactions between individuals of different species scale up to organize ecological communities [1], [2]. The study of food webs, which are networks of trophic interactions among species, has benefited from the proposal of probabilistic, topological models that are able to reproduce the structure of trophic interactions by incorporating simple ecological processes (reviewed by Stouffer [3]). These models offer a way to build realistic food webs using a few parameters such as the number of interacting species and the number of interactions that can be estimated in the field [4], [5], [6], [7]. By connecting the structure of real food webs with candidate underlying processes, such models provide a basis for investigating the implications of food web organization for ecological dynamics [8], species persistence [9], [10], and ecosystem services [11]. Moreover, differences in how closely each model fits the structure of empirical food webs provide insight into the fundamental rules organizing trophic interactions in ecological systems [7], [12], [13].

The majority of studies on how such models reproduce ecological networks have focused on food webs, but there is an increasing body of theory that relies on probabilistic models to understand the structure of networks formed by other kinds of ecological interactions such as mutualisms [2], [14], [15], [16]. The theoretical background for devising specific models for mutualistic networks stems from the fact that antagonisms and mutualisms differ in their fundamental ecological and evolutionary implications [17], [18]. Additionally, mutualistic networks share some marked structural regularities that differ from antagonistic networks such as food webs [18], [19], [20]. For instance, mutualistic networks are best described as two-mode networks in which there are two sets of nodes (e.g., animals and plants) and there are no interactions among species within the same set [21]; in contrast, food webs are organized into several loosely defined trophic levels [12]. Moreover, mutualistic networks tend to be highly nested, that is, a given species interacts with a subset of the partners of species that have more interactions whereas antagonistic networks have lower degrees of nestedness [18], [19](but see [22]). An additional feature of mutualistic networks is that they exhibit right-skewed distributions of the number of interactions per species [21], whereas in food webs, this skewness is, in general, less pronounced [23].

The well-established differences between food webs and mutualistic networks (e.g., [18], [24]) have been counterbalanced by increasing evidence that ecological networks share some basic similarities. For instance, modularity, which was previously predominantly related to antagonistic networks [25], [26], was reported in a large set of mutualistic networks [27]. Along the same lines, although nestedness is often higher in mutualistic than in antagonistic two-mode networks [18], a recent study [22] showed that food webs are actually composed of interconnected, nested, two-mode sub-webs.

Another way in which mutualistic networks and food webs converge is that most mutualistic relationships are, in fact, rooted in consumer-resource relationships [28], [29]. For example, pollination is a type of mutualism that often involves animals foraging for resources provided by flowering plants [30]. Similarly, the frugivores that disperse seeds away from parental trees are usually foraging on fruit pulp [31]. Therefore, even though food webs and mutualistic networks differ in some key aspects of their structure, we should expect that ecological processes related to resource use partially shape these interactions in similar ways. In fact, all of the models proposed for food webs are inspired by processes shaping the consumer-resource interactions in a given locality. These consumer-resource interaction rules are quite general and may also apply to other types of interactions. In this sense, we hypothesize that food web models are able to reproduce the structure of mutualistic networks. To test this hypothesis we adapted food web models to reproduce two-mode networks and compared their performance, and that of a model designed for mutualisms, in reproducing real mutualistic networks. We first calculated summary statistics that described the structural properties of real food webs and used a likelihood-based model selection approach [32] in which we computed the likelihood of obtaining the observed values under a set of candidate network models. Finally, we explored whether simple topological features of mutualistic networks explain the performance of network models.

## Methods

### The models

To test the performance of food web models in reproducing the structure of mutualistic networks, we compiled a set of 10 pollination and 15 frugivory networks totaling 25 mutualistic networks (see Table S1 in supporting information). These networks ranged from networks with small species richness (animal species richness, *A* = 14; plant species richness, *P* = 11) to species-rich networks (*A* = 64; *P* = 43) and from loosely connected networks (connectance, *C* = 0.07) to highly connected networks (*C* = 0.47). For each of those networks, we generated an ensemble of 1000 matrices using four different models to test model performance. Whenever a model generated a network with disconnected species or with a *C* value 3% larger or smaller than the real one, we discarded that network before running the model again [5], [33].

In most mutualistic relationships, interactions can only occur between species in two well-defined sets (e.g., animals and plants), but food webs do not have this two-mode structure. In this sense, in food web models, all species but producers can be both predator and prey; in contrast, animals in the mutualistic networks studied here (pollination and seed dispersal) act as foragers by feeding on fruits and nectar provided by plants. Therefore, we adapted all food web models used to the two-mode nature of mutualistic networks. Our objective was to make as few changes as possible in the original models. We used the same set of simple rules of food web models, but interactions only occurred among species of different sets. As a result, all of the models used the input parameters *A* and *P* as well as the connectance, which is defined as *C* = *E*/*AP*_,_ where *E* is the number of recorded interactions. Below, we first describe each model in detail and then the adaptations we made to deal with the two-mode nature of mutualistic networks. We recognize that the models used in this study only represent a subset of the available food web models (e.g., [6], [13], [34], [35]), but we consider this to be a representative set of models that encompass a wide range of candidate rules for how food webs are built up. Moreover, several models were proposed to explain the structure of mutualistic networks (e.g., [2], [14], [36]). However, because our focus is to build a bridge between models describing antagonistic and mutualistic relationships, we chose to compare food web model performance with that of a recent proposed model that was directly inspired by food web models and has been shown to successfully reproduce the structure of mutualistic networks [15].

#### The cascade model

**Figure 1:**
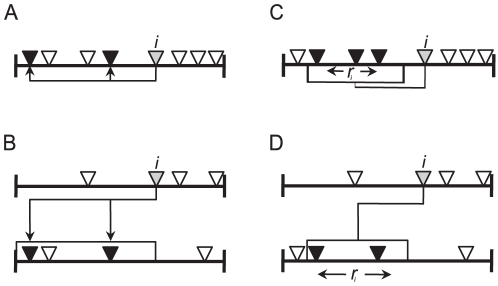
Diagrams comparing original food web models and their two-mode version. (A) the cascade model: each species (represented as an inverted triangle) is assigned a random value being placed along an axis. A given species *i* (gray) potentially interacts with those species whose values are less than the value assigned to *i* (as indicated by arrows); (B) the cascade model for two-mode networks: species that pertain to different sets (e.g. plants and animals) are randomly placed along two separate axes. The upper axis represents the axis of consumers. Therefore a given species *i* in the upper axis potentially interacts with those species in the lower axis whose values are lower than the value assigned to *i*. (C) The niche model: Each species is assigned a random value *n_i_* and consume all species within a range of niche values *r_i_*. (D) The niche model for two-mode networks: species that pertain to different sets (e.g. plants and animals) are placed along two separate axes according to their *n_i_*. Each species in the upper axis consume all species in the lower axis that fall within a range of niche values *r_i_*.

The cascade model was the first of a series of static models that were capable of reproducing some of the structural properties of real food webs [4]. The cascade model is based on the assumption of hierarchical feeding, assigning each of the *S* species in the community a random value that is uniformly drawn from the interval [0,1], which represents species position along a one-dimensional feeding hierarchy (Fig. 1A). Each species has a probability *q* = 2*CS*/(*S* – 1) of consuming those species whose values are smaller than its own [5]. In our effort to adapt the cascade model to the two-mode nature of mutualistic networks, the position of species are assigned independently for animals and plants so that instead of ordering all species along an axis there are two axes: one for animals and the other for plants (Fig. 1B). Animals can potentially interact with plants whose values are smaller than their own but can never interact with other animals. The probability *q* of the original model was not valid for the two-mode version; we defined it as *q = E/T*, in which *T* is the number of possible interactions after species positions are defined. This approach ensures that the model creates networks with connectance that closely resembles the connectance of the empirical food web.

#### The niche model

The niche model addresses some of the limitations of the cascade model; in particular, it addresses the lack of feeding cycles and cannibalism. However, the niche model retains much of the simplicity and tractability embodied by the earlier model [5], [12]. As in the cascade model, the original niche model [5] assigns a position (*n_i_*) taken from a uniform distribution on the interval [0,1] for all *S* species and places each of them along a gradient (Fig. 1C). For each consumer *i*, a niche range *r_i_* = *xn_i_*, where 0 ≤ *x* ≤ 1 is a random variable with a beta-distributed probability density function *p*(*x*) = *β*(1 – *x*)( *β* – 1) with *β* = (1/2*C*) – 1 is then defined. This causes species with higher *n_i_* to tend to eat more species and ensures that the average of all species' *r* equals *C* [33]. The range center (*c_i_*) is a uniformly random number between *r_i_*/2 and min (*n_i_*, 1−*r_i_*/2). A consumer *i* eats all species *j* whose *n_j_* fall within its range (Fig. 1C). Hence, a diet interval *I*(*D_i_*) = [*c_i_−r_i_/2, c_i_+r_i_/2*] is defined for all species. As in the cascade model, to adapt the niche model to mutualistic networks, we defined *n* for plants and animals within two separate axes and diet ranges were defined only for animals and projected in the plants axis, such that animals always behaved as consumers and plants always behaved as food resources (Fig. 1D). Although we recognize that in many cases plant traits are responsible for selecting their interaction partners and thus network assembly could occur from the perspective of plants (e.g., [36]) we opted for an approach that is similar to the original models in which basal species have no defined ranges [5]. To obtain *I*(*D*) for animals, we used functions that are identical to those used in the original model (see Text S1 for reasoning).

In addition to having a more complex set of rules, the niche model differs from the cascade model because it imposes intervality in how links to resource species are assigned. Intervality means that all of the species in a food web can be placed in a fixed order on a line such that each consumer's set of resources forms a single contiguous segment of that line. Therefore, intervality suggests that trophic niche space can be represented by a single dimension [12], [37].

#### The minimum potential model

Even though the niche model seemed to perform fairly well in reproducing most of the features of empirical food webs, food webs often do not show intervality for all species [34]. The minimum potential niche model [7] is a niche-based model that relaxes the interval feeding constraint of the niche model in a similar way to the relaxed niche model [33]. In the minimum potential niche model (hereafter MPN), forbidden interactions lead to gaps in consumers' diets [7]. The MPN model can be seen as a way of embedding multidimensional niches into a one-dimensional context [7]. The MPN model is similar to the niche model in that at first, the positions along the niche axis and diet interval *I*(*D_i_*) of each species are defined. However a consumer eats species that fall within its diet interval with probability 1 – *f*, where *f* is the probability of having forbidden links in the diet (see Text S2). To adapt the MPN model to mutualisms two axes are defined and only animals posses *I*(*D*) as in the niche model.

#### Model of bipartite cooperation networks

The model of bipartite cooperation (hereafter the BC model) was conceived for two-mode cooperation networks such as mutualistic networks and was directly inspired by food web models [15]. Here, we used a slightly different version of the model described by Saavedra et al. [15], following the authors' suggestion. In this model, plants are treated as members of class *P* and animals as members of class *A*. The model consists of two mechanisms: specialization and interaction. The specialization rule determines the number of interaction partners, *l_pi_*, of each species *p* ∈ *P*. This number is determined by the interaction among two values: the reward trait, *t_Rp_*, a number randomly drawn from an uniform distribution [0,1], which is attenuated or amplified by an external factor *λ_p_* that is randomly drawn from an exponential distribution, which accounts for effects such as population density. The higher the reward value of plant *p_i_*, the higher is the number of potential interactions established by *p_i_*. The interaction rule determines which species *a* ∈ *A* interacts with each species *p* ∈ *P*. Interactions are limited by the complementarity between the reward traits, *t_Rp_*, for *p* ∈ *P* and foraging traits, *t_Fa_*, for *a* ∈ *A*. The foraging trait *t_Fa_*_,_ which are also uniformly drawn from [0,1], limits the range of possible partners for each member of class *A*, but again, interactions are affected by external factors *λ_lp,_* which could represent, for instance, temporal variation and population density that are randomly drawn from an exponential distribution for each interaction.

Interactions are distributed to plants sequentially, in ascending order, according to their foraging traits *t_Rp_*. Whenever *t_Rpi_*>*λ_lpi_* each link *l_pi_* is connected to the first node *a′* ∈ *A′,* where *A′* is the subset of nodes in *A* that have not already been linked to by another node *p* ≠ *p_i_*. Conversely, if *t_Rpi_* ≤ *λ_lpi_*_,_ interactions of *p_i_* are distributed using a mechanism similar to that proposed by Cattin et al. [6], i.e., a plant *p* ∈ *P* with lower trait value is randomly selected, and an interaction is established with an animal randomly chosen among its partners *a*'' ∈ *_A_*'' where *A*'' is the subset of nodes in *A* that have been linked in a previous time step. If the supply of nodes in either *A*′ or *A*'' is exhausted before all *l_pi_* links have been allocated, then nodes in the other subset are linked to instead. For additional detailed information on the model we refer readers to Saavedra et al. [15].

### Performance analysis

For each empirical network and their theoretical counterparts, we calculated four structural properties often used to describe the structure of mutualistic networks: the degree of nestedness [19], degree of modularity [27] and the cumulative degree distributions for both animals and plants [21]. We then used two procedures, model fit and model likelihood, to evaluate the model performances in reproducing these structural properties. Below, we describe each structural property and both procedures to test model performances.

#### Nestedness

Nestedness is a property of networks in which the interacting assemblage of a species is a subset of the interacting assemblage of species with more interactions [19]. The index *NODF* (an acronym for nestedness metric based on overlap and decreasing fill [38]) was used to compute the degree of nestedness of both empirical networks and those generated by the models. *NODF* ranges from 0, when the matrix shows other nonrandom patterns of resource use, to 100, when the matrix is perfectly nested (additional information on *NODF* at [38]).

#### Modularity

Modules within a network are subsets of species that are more densely connected to each other than to species in other modules [39]. To find the best partition of a given network into modules, we used the simulated annealing algorithm to maximize and index of modularity, *M*, that accounts for the number of interactions between species belonging to the same module and the number of interactions between species belonging to different modules [39]. *M* equals 0 if nodes are placed at random into modules or if all nodes are in the same module and approaches 1 if modules have well-delimited boundaries (i.e., few between-module interactions). Although *M* does not take into account the fact that mutualistic networks are two-mode networks, any potential effect of the two-mode structure on modularity is controlled since all networks analyzed have two sets of species. Thus, any difference in *M* among real and theoretical networks cannot be related to the two-mode structure.

#### Degree distributions

The degree, *k*, of a species *i* in a mutualistic network can be defined as the number of species with which species *i* interacts. Therefore, the cumulative degree distribution of a mutualistic network describes the proportion of species with *k* or more interaction partners [21]. It can therefore be considered a description of the pattern of ecological specialization in the community [40]. Because we dealt with two-mode networks, degree distributions were calculated separately for animals and plants.

#### Model fit

To test whether the models were capable of reproducing empirical network properties, we used different procedures depending on the topological property analyzed. For nestedness and modularity, we calculated the normalized model error (*NME*) between the empirical values and the values obtained from the numerical simulations of each model. The *NME* can be defined as the difference between the model's median property value and the empirical value divided by the difference between the model's median property value and the property value at the 2.5% or 97.5% quantiles, depending on whether the empirical value is lower or larger than the model's median [33]. A value of *NME* greater than 1 means that the empirical value is significantly different from the degrees of nestedness or modularity of networks generated by a given model [33]. By doing this, we did not make particular assumptions about the distribution of property values generated by the food web model [33]. Here, we used a slightly modified version of *NME* in which we use the absolute value of the difference between the median and the quantile to normalize the index so that the direction of the deviation is maintained. Therefore, a positive *NME* indicates overestimation of a property value by the model, and a negative *NME* indicates underestimation. To test whether the models were capable of reproducing degree distributions, we used the Kolmogorov-Smirnov test [13].

#### Model likelihood

The procedures described above allow us to distinguish among situations in which a network property is reproduced or not. However one model could be regarded as the one with larger fit when in fact it just produces a larger variance of metric values. Therefore, to perform comparisons among models, we opted to use the likelihood approach, which is a statistical framework specifically designed to allow direct comparisons among many competing models [32]. Recent studies (e.g., [41], [42]) aiming to describe how mutualistic networks change over time have shown that species pairwise-interactions are highly variable whereas the overall network structure often remains unmodified. Therefore, we opted for a likelihood approach that differs from recent proposed likelihood frameworks, which focused on finding the model that was most likely to reproduce all pair-wise interactions observed in real networks [7], [43]. Because we were interested in the distinct overall structural properties of each network, the objective of our likelihood approach is to determine which model was most likely to reproduce the observed value for each property separately, gauged by a summary statistic (see [44]). If the difference between the negative log-likelihood of the best model and another given model was less than 2, they were considered equally plausible [32]. For additional information on how we computed model likelihood using simulations see Text S3.

#### Correlates of model performance

To develop a better understanding on which characteristics of the real network affects the performance of each model, we used a general linear model to test whether features such as connectance (*C*), animal species richness (*A*), plant species richness (*P*), and the nestedness and modularity values themselves affected the normalized errors of each model, *NME* (i.e., a proxy for the degree of fit of a given model for each real network). We used relative nestedness (N*; [19]) and relative modularity (M*), in which the observed value is corrected using the average value of 1000 random networks with the same size and connectance as the original network. The results still held if we assumed other theoretical benchmark that kept heterogeneity in the number of interactions across species (“null model 2”, [19], Table S2). There was no correlation among N* and M* (*r* = −0.39, *n* = 25, *P*>0.05), which allowed both to be included in the analysis as explanatory variables. Then, for each of the four models, we used multiple regression models of the following form:

NME  =  β_0_ + C×β_1_ + A×β_2_ + P×β_3_ + N*×β_4_ + M*×β_5_ + ε

where *NME* is the normalized error, *β_i_* are the coefficients of the multiple regression and *ε* is the usual Gaussian error. All regressions assumptions, such as the normality of residuals, were met. Then we used the Akaike criterion to select the best set of variables in predicting *NME* [45]. The tests were performed for *NME*s in reproducing *NODF* and *M* separately.

## Results

All models performed remarkably well in reproducing both the nestedness and modularity of the mutualistic networks. The percentage of networks whose metrics were reproduced by each model varied between nearly 50% and 95% (Table 1). The models that reproduced the properties in the largest proportion of networks were the two-mode cascade model and the BC model (Table 1). When we directly compared the models as competing hypotheses using the likelihood approach, the outcome of the model comparison depended on the property being analyzed (Table 1). The cascade and niche models were among the most likely models for 84% of the networks considering nestedness. This result held when using a different nestedness metric, the matrix temperature, which indicates that these results are not affected by metric choice (Text S4). Similarly, when considering modularity, the cascade model was among the most likely models for 84% of the networks. However, the BC model instead of the niche model was the second best model in terms of reproducing modularity (Table 1). Regarding degree distributions, the results are less straightforward. All four models reproduced degree distributions for nearly all analyzed networks according to the Kolmogorov-Smirnov test results (Table 1). Nonetheless, the model comparison suggested that the cascade model was usually among the best models in reproducing plants degree distributions, whereas the niche and BC models outperformed the others more often in reproducing the degree distribution of animals (Table 1).

**Table 1:** Proportion of mutualistic networks (*N* = 25) whose properties were reproduced by each model (*NME*<1; *P_KS_*<0.05)/proportion of networks in which each model was among the most likely.

	*NODF*	*M*	*Pk_A_*	*Pk_P_*
Cascade	0.84/0.84	0.88/0.84	0.96/0.52	1.00/0.76
Niche	0.80/0.84	0.52/0.44	1.00/0.88	0.96/0.60
MPN	0.60/0.68	0.56/0.60	0.84/0.64	1.00/0.64
BC	0.72/0.80	0.80/0.72	0.96/0.84	0.92/0.60

Columns represent the network properties analyzed: *NODF*  =  nestedness, *M*  =  modularity, *Pk_A_*  =  cumulative degree distribution of animals, *Pk_P_*  =  cumulative degree distribution of plants. Because more than one model could reproduce or be among the most likely models in reproducing the property of a given network the sum of the proportions in each column is larger than 1.

**Figure 2:**
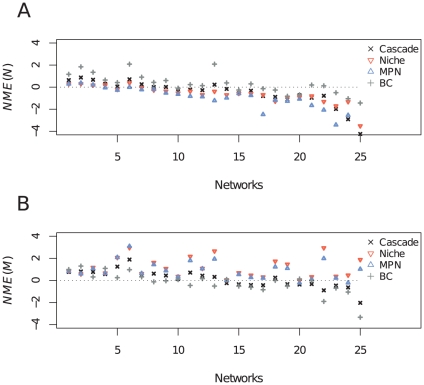
Normalized error (*NME*) of each model in reproducing nestedness (A) and modularity (B) for each of the 25 analyzed networks. In (A) networks are sorted in increasing order of relative nestedness. Notice nestedness tend to be underestimated for networks with large nestedness degrees as suggested by partial regression coefficients (Table 2). In (B) networks are sorted in increasing order of relative modularity.

The sign of *NME* indicates whether the model overestimates or underestimates a property value for a given network. Therefore, an excess of negative values of *NME* indicates that a model often underestimates a given property, whereas positive values suggest that the model has a tendency to overestimate it. The niche and MPN models tended to generate networks with lower degrees of nestedness and higher degrees of modularity than real networks (Fig. 2). The cascade and BC models were more balanced and showed fewer signs of systematic biases in one direction or another (Fig. 2). However, the degree of fit of models was associated with basic topological features of networks (see Table 2). Noteworthy network basic features explained between 70 and 95% of variation in model fit regarding nestedness and modularity. All models tended to underestimate nestedness as the degree of relative nestedness observed increased (*P*<0.01; Table 2, Fig. 2A). The degree of relative nestedness also affected the ability of the cascade, niche and MPN models to reproduce modularity. These models tended to overestimate network modularity for networks that had a high degree of relative nestedness (Table 2). The degree of relative modularity had the opposite effect for the cascade, MPN and BC models. When reproducing networks with high relative modularity, these models were more prone to underestimate modularity (Table 2). Connectance also affected model fit. Networks with larger connectance tended to have their degrees of modularity and nestedness overestimated by the cascade (only for nestedness) and BC models, whereas modularity *NME* decreased with increasing connectance for the niche model (Table 2).

**Table 2:** Effects of basic real network features in model degree of fit as expressed by the *NME*.

	*F*	*df*	*r^2^*	*A*	*P*	*C*	*N* *	*M* *
Cascade	266.4**	23, 1	0.92	–	–	–	−2.01***	–
Niche	67.1***	21,3	0.89	−0.01*	–	–	−1.05***	2.07*
MPN	241.5***	22,2	0.95	−0.01***	–	–	−1.84***	–
BC	19.11***	21,3	0.70	–	–	3.15**	−0.6***	4.12*
Cascade	31.54**	20,4	0.83	0.01*	–	1.6*	0.60***	−6.15***
Niche	40.5***	21,3	0.83	0.01**	–	−1.59*	0.76***	–
MPN	86.84***	22,2	0.87	0.01***	–	–	1.00***	–
BC	31.89***	22,2	0.72	–	–	2.53**	–	−8.34***

Multiple regression analyses results reporting the F-statistics (*F*), degrees of freedom (*df*), determination coefficient (*r^2^*) and the partial regression coefficients of each of the following factors: animal species richness (*A*), plant species richness (*P*), connectance (*C*), relative nestedness (N*) and relative modularity (M*). Traces mean that the factor was not included in the best regression. The significance of each factor and the model as a whole is represented as follows:

*<0.05;

**<0.01;

***<0.001. The first 4 rows correspond to the *NME* for nestedness and the last for modularity.

## Discussion

Our results show that all four models performed fairly well in reproducing the properties of empirical mutualistic networks. However, the cascade and BC models more often generated theoretical networks that were in agreement with the structure of real mutualistic networks. Moreover, the cascade model was frequently among the most likely candidate models in reproducing the structure of mutualistic networks. Although the performance of the cascade and BC models was similar, the cascade model is much simpler than the BC model. In addition to attributing a value to each species as done in the cascade model, the BC model has many other free parameters that act as external factors that affect interactions. Therefore, the good performance of the cascade model appears even better when model complexity is taken into account.

In food webs, the cascade model also reproduced some aspects of the structure of interactions between consumers and resources [5]. Nevertheless, other models such as the niche and MPN models often outperformed the cascade model in reproducing food web structure [5], [7], [33]. The niche model was mainly proposed as a solution that included the possibilities of feeding loops and cannibalism, which were not allowed by the minimal rules of the cascade model [12]. In plant-animal mutualisms, on the other hand, interactions only occur between species in different trophic levels (plants and animals that forage in plant resources). Therefore, as we dealt with this two-mode structure of mutualisms, feeding loops were not a problem. This may partially explain the success of the cascade model for mutualisms in spite of being outperformed by niche model derivatives in the context of food webs [5], [7], [33]. In addition to the two-mode structure, other biological aspects of mutualisms might explain why the strict feeding hierarchy generated by the cascade model suffices to reproduce much of the structure of mutualistic networks.

Hierarchy is also an essential component in the BC model, which was directly inspired by the set of rules of food web models [15]. The success of the BC model in reproducing network structural patterns in a previous work [15] already suggested that such hierarchical processes should play a crucial role in organizing mutualistic networks. Because all models considered here encompass hierarchical processes our results reinforce their relevance in mutualisms. Moreover, the similar success in reproducing the structure of real networks of both BC and the much simpler food web models suggest that the feeding hierarchy by itself is enough to capture much of the structure of mutualistic networks. Although multiple processes may generate similar patterns in ecological systems, our results at least indicate possible mechanisms shaping the organization of mutualistic interactions in networks of interacting species.

The most compelling biological basis proposed for the ordering dimension that induces a feeding hierarchy in food web models is body size [12], [37], [43], [46]. In this sense, in the context of food webs, the hierarchical ordering in the cascade model would lead to larger species interacting with smaller species. Similarly, in niche models, larger species would tend to have wider trophic niches [43]. In the case of pollination and frugivory networks, such hierarchy could refer to any measurable traits related to the feeding interaction among fruiting/nectar-producing plants and fruit/nectar consumers such as bill diameter, bill or mouthparts length, and fruit size or corolla depth. Such traits would be represented in the adapted models as the two independent axes in which animals and plants are ordered. Indeed in a series of studies, Stang et al. [47], [48] showed that structural patterns of pollination networks such as nestedness could be reproduced by incorporating size thresholds imposed by floral morphology on nectar-feeding animals. Moreover, body size was found to predict the number of interactions of ants in ant-plant mutualisms [49]. Finally, larger frugivores are often able to eat a large variation in fruit sizes than smaller frugivores, leading to hierarchical ordering in frugivory [31]. From an evolutionary perspective trait based feeding hierarchies can emerge as a consequence of natural selection favoring particular high profitable resource combinations [17].

The way each model encompass feeding hierarchies may also partially explain differences in model performance. Species-rich mutualisms often form networks modules of interacting species based on shared phenotypic traits such as fruit color, flower shape, animal body mass [27], [50]. Nevertheless, modularity in mutualisms such as pollination and seed dispersal is often smaller than observed in antagonistic interactions [18] or in symbiotic mutualisms [2]. The strict feeding hierarchy imposed by the cascade model causes high overlap in the set of interaction partners among consumer species, leading to low modularity. Conversely the set of rules in other food web models, such as niche and MPN models, that partially relax the cascade hierarchy [9] might favor higher modularity. In niche and MPN model, species whose feeding ranges overlap may form network modules that differ from modules formed by species whose feeding ranges overlap farther in the niche axis. In fact, both niche and MPN models were outperformed by the cascade and BC models in reproducing the low degree of modularity in mutualistic networks, especially because they usually generated networks that were more modular than the empirical ones. This may also partially explain the superior performance of both the niche and MPN models in comparison with the cascade model in generating the more modular structure of food webs [5], [33].

The degree of relative nestedness and relative modularity of the real network were the main features of real networks affecting model fit; for networks with higher relative nestedness, the cascade, niche and MPN models tended to underestimate nestedness and overestimate the modularity of real networks. Conversely for networks with higher relative modularity, real modularity was usually underestimated. The sensitivity of the models accuracy to the degree of nestedness and modularity in the real networks indicates that the high degrees of nestedness or modularity observed in some mutualistic networks are not completely explained by the processes incorporated in food web models analyzed here. Stouffer et al. [13] showed analytically that a food web model should satisfy two criteria in order to reproduce most empirical food web properties: niche values should form a totally ordered set, and each species has a specific, exponentially decaying probability of preying on a fraction of the species with lower niche-values. In the context of mutualisms, it seems that a model's ability to reproduce empirical networks is not only a matter of reproducing the functional forms for the distributions of numbers of prey, predators and links per species, but also of reproducing the relationship between nestedness and modularity. Many mechanisms have been proposed for the occurrence of the nested pattern, namely, differences in abundance among species [26], [51], low interaction intimacy [2], trait complementarity and/or exploitation barriers coupled with coevolutionary convergence [14], [17], [48] and frequent extinctions of specialist-specialist interactions [52]. Along the same lines, trait matching along with phylogenetic constraints [20] and high interaction intimacy [2] are regarded as the main mechanisms that could lead to a modular structure in mutualistic networks [27]. The rules of the cascade, niche and MPN models can be interpreted as a form of encompassing trait complementarity and exploitation barriers among interacting species. Similarly, the BC model is based on the complementarity among plants reward traits and animals foraging traits. Although they do incorporate complementarity, they do not explicitly consider other mechanisms shaping network structure such as interaction intimacy, differential extinction and phylogenetic constraints. Evolving network models, models in which the number of species and interactions change over time, have also been shown to partially explain the structure of mutualistic networks [2], [36]. Future studies combining the mechanisms present in these two different classes of models might provide additional insights in the organization of mutualistic networks.

To sum up, food web minimal models were capable of reproducing most of the mutualistic networks analyzed. Noteworthy, even the cascade model, the simplest among the models considered here, reproduced the structure of nearly the whole set of networks. Such results open the possibility that the assembly of networks that describe mutualisms and antagonisms obey a similar simple set of rules and reinforce that feeding hierarchy might be a fundamental piece in this puzzle. Therefore, despite the differences in ecology and evolution of mutualisms and antagonisms [17], [18], they seem to share some key aspects. Our knowledge of the assembly of natural communities would benefit from future studies that scrutinize those commonalities and differences and attempt to sort out the evolutionary and ecological mechanisms that are responsible for each.

## Supporting Information

Text S1 The probability distribution for *X* for niche and MPN models.
(DOC)

Text S2 Definition of *f* for the MPN model.
(DOC)

Text S3 Computing model likelihood.
(DOC)

Text S4 Results using the metric Nestedness Temperature (*T*).
(DOC)

Table S1 Information on analyzed networks.
(DOC)

Table S2 Effects of basic real network features in *NME* with different N* and M* calculations.
(DOC)

## References

[1] Dupont YL, Trøjelsgaard K, Olesen JM (2010). Scaling down from species to individuals: a flower-visitation network between individual honeybees and thistle plants.. Oikos.

[2] Guimarães PR, Rico-Gray V, Oliveira P, Izzo T, dos Reis SF (2007). Interaction intimacy affects structure and coevolutionary dynamics in mutualistic networks.. Curr Biol.

[3] Stouffer DB (2010). Scaling from individuals to networks in food webs.. Funct Ecol.

[4] Cohen JE, Briand F, Newman CM (1990). Community food webs: data and theory..

[5] Williams RJ, Martinez ND (2000). Simple rules yield complex food webs.. Nature.

[6] Cattin M-F, Bersier L-F, Banašek-richter C, Baltensperger R, Gabriel J-P (2004). Phylogenetic constraints and adaptation explain food-web structure.. Nature.

[7] Allesina S, Alonso D, Pascual M (2008). A general model for food web structure.. Science.

[8] Brose U, Jonsson T, Berlow E, Warren P (2006). Consumer-resource body-size relationships in natural food webs.. Ecology.

[9] Dunne JA, Williams RJ (2009). Cascading extinctions and community collapse in model food webs.. Phil Trans R Soc B.

[10] Gross T, Rudolf L, Levin SA, Dieckmann U (2009). Generalized models reveal stabilizing factors in food webs.. Science.

[11] Montoya JM, Rodríguez MA, Hawkins BA (2003). Food web complexity and higher-level ecosystem services.. Ecol Lett.

[12] Dunne JA, Pascual M, Dunne JA (2006). The network structure of food webs.. Ecological networks: linking structure to dynamics in food webs.

[13] Stouffer DB, Camacho J, Guimerà R, Ng CA, Amaral LAN (2005). Quantitative patterns in the structure of model and empirical food webs.. Ecology.

[14] Santamaría L, Rodríguez-Gironés MA (2007). Linkage rules for plant–pollinator networks: trait complementarity or exploitation barriers.. PLoS Biol.

[15] Saavedra S, Reed-Tsochas F, Uzzi B (2009). A simple model of bipartite cooperation for ecological and organizational networks.. Nature.

[16] Campbell C, Yang S, Albert R, Shea K (2011). A network model for plant-pollinator community assembly.. Proc Natl Acad Sci U S A.

[17] Thompson JN (2005). The geographic mosaic of coevolution..

[18] Thébault E, Fontaine C (2010). Stability of ecological communities and the architecture of mutualistic and trophic networks.. Science.

[19] Bascompte J, Jordano P, Melián CJ, Olesen JM (2003). The nested assembly of plant - animal mutualistic networks.. Proc Natl Acad Sci U S A.

[20] Vázquez DP, Blüthgen N, Cagnolo L, Chacoff NP (2009). Uniting pattern and process in plant–animal mutualistic networks: a review.. Ann Bot-London.

[21] Jordano P, Bascompte J, Olesen JM (2003). Invariant properties in coevolutionary networks of plant–animal interactions.. Ecol Lett.

[22] Kondoh M, Kato S, Sakato Y (2010). Food webs are built up with nested subwebs.. Ecology.

[23] Dunne JA, Williams RJ, Martinez ND (2002). Food-web structure and network theory: The role of connectance and size.. Proc Natl Acad Sci U S A.

[24] Bascompte J, Jordano P (2007). Plant-animal mutualistic networks: the architecture of biodiversity.. Ann Rev Ecol Evol Syst.

[25] Prado PI, Lewinsohn TM (2004). Compartments in insect–plant associations and their consequences for community structure.. J Anim Ecol.

[26] Lewinsohn TM, Prado PI, Jordano P, Bascompte J (2006). Structure in plant–animal interaction assemblages.. Oikos.

[27] Olesen JM, Bascompte J, Dupont YL, Jordano P (2007). The modularity of pollination networks.. Proc Natl Acad Sci U S A.

[28] Ings TC, Montoya JM, Bascompte J, Blüthgen N, Brown L (2009). Ecological networks – beyond food webs.. J Anim Ecol 78: 253-.

[29] Holland JN, DeAngelis DL (2010). A consumer-resource approach to the density-dependent population dynamics of mutualism.. Ecology.

[30] van der Pijl L (1982). Principles of dispersal in higher plants. 3rd edn..

[31] Jordano P, Fenner M (2000). Fruits and frugivory.. Seed: the ecology of regeneration in plant communities. 2nd ed.

[32] Royall R (1997). Statistical evidence: a likelihood paradigm..

[33] Williams RJ, Martinez ND, Lake BB (2008). Success and its limits among structural models of complex food webs.. J Anim Ecol.

[34] Stouffer DB, Camacho J, Amaral LAN (2006). A robust measure of food web intervality.. Proc Natl Acad Sci U S A.

[35] Allesina S, Pascual M (2009). Food web models: a plea for groups.. Ecol Lett.

[36] Takemoto K, Arita M (2010). Nested structure acquired through simple evolutionary process.. J Theor Biol.

[37] Zook AE, Eklof A, Jacob U, Allesina S (2011). Food webs: ordering species according to body size yields high degree of intervality.. J Theor Biol.

[38] Almeida-Neto M, Guimarães P, Guimarães PR, Loyola RD, Ulrich W (2008). A consistent metric for nestedness analysis in ecological systems: reconciling concept and measurement.. Oikos.

[39] Guimerà R, Amaral LAN (2005). Functional cartography of complex metabolic networks.. Nature.

[40] Olesen JM, Jordano P (2002). Geographic patterns in plant-pollinator mutualistic networks.. Ecology.

[41] Petanidou T, Kallimanis AS, Tzanopoulos J, Sgardelis SP, Pantis JD (2008). Long-term observation of a pollination network: fluctuation in species and interactions, relative invariance of network structure and implications for estimates of specialization.. Ecol Lett.

[42] Díaz-Castelazo C, Guimarães PR, Jordano P, Thompson JN, Marquis RJ (2010). Changes of a mutualistic network over time: reanalysis over a 10-year period.. Ecology.

[43] Williams RJ, Anandanadesan A, Purves D (2010). The probabilistic niche model reveals the niche structure and role of body size in a complex food web.. PloS one.

[44] Hartig F, Calabrese JM, Reineking B, Wiegand T, Huth A (2011). Statistical inference for stochastic simulation models - theory and application.. Ecol Lett.

[45] Burnham KP, Anderson DR (2002). Model selection and multimodel inference: a practical information-theoretic approach..

[46] Woodward G, Ebenman B, Emmerson M, Montoya JM, Olesen JM (2005). Body size in ecological networks.. Trends Ecol Evol.

[47] Stang M, Klinkhamer PGL, van der Meijden E (2007). Asymmetric specialization and extinction risk in plant-flower visitor webs: a matter of morphology or abundance?. Oecologia.

[48] Stang M, Klinkhamer PGL, Waser NM, Stang I, van der Meijden E (2009). Size-specific interaction patterns and size matching in a plant-pollinator interaction web.. Ann Bot-London.

[49] Chamberlain SA, Holland JN (2009). Body size predicts degree in ant-plant mutualistic networks.. Funct Ecol.

[50] Donatti C, Guimarães PR, Galetti M, Pizo MA, Marquitti FMD (2011). Analysis of a hyper-diverse seed dispersal network: modularity and underlying mechanisms.. Ecol Lett.

[51] Krishna A, Guimarães PR, Jordano P, Bascompte J (2008). A neutral-niche theory of nestedness in mutualistic networks.. Oikos.

[52] Ollerton J, Mccollin D, Fautin DG, Allen GR (2007). Finding NEMO: nestedness engendered by mutualistic organization in anemonefish and their hosts.. Oecologia.

