# Supplementary material for: Do Food Web Models Reproduce the Structure of Mutualistic Networks?
Source: PLoS One. 2011 Nov 2;6(11):e27280. doi: 10.1371/journal.pone.0027280 (PMC3206955; doi:10.1371/journal.pone.0027280)
Supplement: Text S1 — The probability distribution for X for niche and MPN models. (DOC) [file pone.0027280.s001.doc]

Supporting information for “Do food web models reproduce the structure of mutualistic networks?” by MM Pires, PI Prado, PR Guimarães Jr.

**Text S1**. The probability distribution for *X* for niche and MPN models

The distribution of *X,* which is used to obtain ranges for animals in the niche model, need to be chosen so that the expected value of this probability matches the empirical value of connectance *C* [1]. As in the original niche model we defined *X* as a random variable that is beta distributed with parameters (1, *β*). Therefore the theoretical model connectance is given by:

Because the density of species on the segment containing plants equals the number of plants *P,* each animal *A* will have on average *E*[*r_i_*]*P* interactions. This procedure is repeated for all animals so that the total expected number of connections is:

Because *E*[*L*] should be equal to *C***A***P*, which is the number of interactions observed in the real network, *β* is given by:

the same equation used in the original model [2].

**References**

1. Allesina S, Alonso D, Pascual, M (2008) A general model for food web structure. Science 320: 658-661.

2. Williams RJ, Martinez ND (2000) Simple rules yield complex food webs. Nature 404: 180-183.
