# Supplementary material for: Do Food Web Models Reproduce the Structure of Mutualistic Networks?
Source: PLoS One. 2011 Nov 2;6(11):e27280. doi: 10.1371/journal.pone.0027280 (PMC3206955; doi:10.1371/journal.pone.0027280)
Supplement: Text S2 — Definition of f for the MPN model. (DOC) [file pone.0027280.s002.doc]

Supporting information for “Do food web models reproduce the structure of mutualistic networks?” by MM Pires, PI Prado, PR Guimarães Jr.

**Text S2** Definition of *f* for the MPN model

The MPN model is similar to the niche model in that at first, the positions along the niche axis and diet range *I*(*D_i_*) of each species are defined [1] However, before the subsequent steps we must first reorganize the matrix describing the real food web, maximizing the ‘‘interval’’ ordering of the food web. This process yields the best approximation to a food web where the species and their diets are organized along a single dimension [2]. To find this most interval ordering, we used the simulated annealing algorithm (SA), which is a stochastic optimization technique (for details on the simulated annealing algorithm, we refer readers to [2,3]). When attempting to find the most interval ordering, the objective is to minimize the discontinuity of all consumers’ resources [2]. Therefore, we used the cost function *G*(*O_k_*) as defined by Stouffer *et al.* [2], which sums up the gaps in all consumers’ diets for a given ordering (*O_k_*) of a food web *A*. Therefore, the optimal ordering is that with the smallest estimated *G*. Our procedure differed from the one described in Stouffer et al. [2] because we only needed to reorder the plant positions in the interaction matrix to reduce discontinuity in the set of interactions of each animal.

After obtaining the maximum interval network, we were able to find *f*. In this model, *f* is the probability of having forbidden links in the diet (i.e., non-prey items that are within the potential niche range of a consumer and therefore reduce intervality). The probability *f* is defined as *f* = *F*/*I*, where *F* is the number of non-prey items within the potential niche range of all species and *I* is the number of potential “inner” prey (i.e., those species in a diet interval that are neither the leftmost nor the rightmost species included in the diet of predator *i*). To obtain both *F* and *I* using the empirical network, we used the maximum interval network. After calculating *f*, interactions were distributed as in the niche model [4] (i.e, a consumer eats species that fall within its diet interval but with probability 1 – *f*).

**References**

1. Allesina S, Alonso D, Pascual M (2008) A general model for food web structure. Science 320: 658-660.

2. Stouffer DB, Camacho J, Amaral LAN (2006) A robust measure of food web intervality. Proc Natl Acad Sci U S A 103: 19015-19020.

3. Guimerà R, Amaral LAN (2005) Functional cartography of complex metabolic networks. Nature 433: 895-900.

4. Williams RJ, Martinez ND (2000) Simple rules yield complex food webs. Nature 404: 180-183.
