# Supplementary material for: Do Food Web Models Reproduce the Structure of Mutualistic Networks?
Source: PLoS One. 2011 Nov 2;6(11):e27280. doi: 10.1371/journal.pone.0027280 (PMC3206955; doi:10.1371/journal.pone.0027280)
Supplement: Text S3 — Computing model likelihood. (DOC) [file pone.0027280.s003.doc]

Supporting information for “Do food web models reproduce the structure of mutualistic networks?” by MM Pires, PI Prado, PR Guimarães Jr.

**Text S3** Computing model likelihood

To calculate model likelihood, we generated the probability density function of nestedness and modularity under each model for each real mutualistic network analyzed. These distributions then gave the probability that each theoretical model attributed to the observed values for the real networks. Because any function that is proportional to these probabilities is a likelihood [1], we took the probabilistic density at the empirical value as the likelihood of the model for each observed matrix. In this way, we used empirical distributions to compute the likelihood. To obtain the probabilistic density function for each model, we computed the kernel density estimates of the values obtained in the 1,000 numerical simulations [2]. We then performed a cubic spline interpolation using these estimates to obtain the probabilistic density of each empirical value [3]. When the empirical value was outside of the range of values generated by the model, we conservatively considered the likelihood as the probabilistic density associated with the most extreme value generated by the model to avoid underestimating a model’s likelihood. Kernel density estimates and interpolation were performed using R software [4].

We used a similar approach to obtain the likelihood that a model generates the degree distribution of animals and plants. First, we estimated the probability that each model had of generating species with a given number of interactions (*k*) as the frequency of species with *k* interactions divided by the number of species in all the 1000 simulations. The log-likelihood of the model in generating the cumulative degree distribution is the sum of the logarithms of the probabilities the model attributes to each *k*. The model with lower negative log-likelihood was considered the best model in reproducing a network property. If the difference between the negative log-likelihood of the best model and another given model was less than 2, they were considered equally plausible.

**References**

1. Edwards AWF (1972) Likelihood - an account of the statistical concept of likelihood and its application to scientific inference. New York: Cambridge University Press. 235 p.

2. Venables WN, Ripley BD (2002) Modern applied statistics with S. 4th edn. New York: Springer. 512 p.

3. Fritsch FN, Carlson RE (1980) Monotone piecewise cubic interpolation. SIAM J Numer Anal 17: 238-246.

4. R Development Core Team (2010) A language and environment for statistical computing. Vienna: R Foundation for Statistical Computing.
