# Supplementary material for: Do Food Web Models Reproduce the Structure of Mutualistic Networks?
Source: PLoS One. 2011 Nov 2;6(11):e27280. doi: 10.1371/journal.pone.0027280 (PMC3206955; doi:10.1371/journal.pone.0027280)
Supplement: Text S4 — Results using the metric Nestedness Temperature (T). (DOC) [file pone.0027280.s004.doc]

Supporting information for “Do food web models reproduce the structure of mutualistic networks?” by MM Pires, PI Prado, PR Guimarães Jr.

**Text S4** Results using the metric Nestedness Temperature (*T*)

Since recent studies have reported differences in the behavior of different nestedness metrics [1,2] we tested whether our results regarding model performance in reproducing nestedness were sensitive to metric choice. To accomplish that we computed matrix temperature, *T* [3], a more traditional measure of nestedness for real and model generated networks and calculated *NME*. Consistent with the results using the index *NODF*, the niche and cascade model reproduced the nestedness (|*NME*| < 1) of 88% and 84 % of real networks when using *T* as nestedness metric, whereas MPN and BC model reproduced nestedness of 76% and 40% of real networks.

**References**

1. Ulrich W, Almeida-Neto M, Gotelli NJ (2009) A consumer’s guide to nestedness analysis. Oikos 118: 3-17.

2. Joppa LN, Montoya JM, Solé R, Sanderson J, Pimm SL (2010) On nestedness in ecological networks. Evol Ecol Res 12: 35-46.

3. Atmar W, Patterson BD (1993) The measure of order and disorder in the distribution of species in fragmented habitat. Oecologia 96: 373-382.
