# Supplementary material for: Do Food Web Models Reproduce the Structure of Mutualistic Networks?
Source: PLoS One. 2011 Nov 2;6(11):e27280. doi: 10.1371/journal.pone.0027280 (PMC3206955; doi:10.1371/journal.pone.0027280)
Supplement: Table S1 — Information on analyzed networks. (DOC) [file pone.0027280.s005.doc]

Supporting information for “Do food web models reproduce the structure of mutualistic networks?” by MM Pires, PI Prado, PR Guimarães Jr.

**Table S1** Information on analyzed networks. *T* = network type (*P* = pollination; *F* = frugivory); *A* = animal species richness; *P* = plant species richness; *C* = network connectance.

| **Reference** | **Locality** | ***T*** | ***A*** | ***P*** | ***C*** |
| --- | --- | --- | --- | --- | --- |
| [1] | Córdon del Cepo, Chile | P | 64 | 43 | 0.07 |
| [1] | Córdon del Cepo, Chile | P | 25 | 36 | 0.09 |
| [2] | Princeton, Mercer, New Jersey, USA | F | 7 | 21 | 0.34 |
| [3] | Mount Missim, 9 km N-NE Wau, Morobe Prov., New Guinea | F | 9 | 31 | 0.42 |
| [4] | Nava de las Correhuelas. S. Cazorla, SE Spain. | F | 33 | 25 | 0.18 |
| [5] | Tropical rainforest. Queensland, Australia. | F | 7 | 71 | 0.28 |
| [6] | Cordillera Cantábrica, N Spain | F | 7 | 12 | 0.47 |
| [7] | Hato Ratón, Sevilla, Spain | F | 17 | 16 | 0.44 |
| [8] | Hazen Camp, Ellesmere Island, Canada | P | 91 | 20 | 0.10 |
| [9] | Kuala Lompat, Krau Game Reserve. | F | 61 | 25 | 0.33 |
| Montero unpubl. | Coastal Bog, Denmark | P | 40 | 10 | 0.18 |
| [10] | Rocky cliff and open herb community, Azores Island | P | 12 | 10 | 0.25 |
| [10] | Coastal Forest, Mauritius Island | P | 13 | 14 | 0.28 |
| [11] | Artic community, Canada | P | 18 | 11 | 0.19 |
| Jordano P unpub. | Nava Noguera, Sierra de Cazorla, SE Spain | F | 28 | 18 | 0.25 |
| [12] | Morant Point, Jamaica | P | 36 | 61 | 0.08 |
| [13] | Gabon, Africa | F | 8 | 19 | 0.49 |
| [14] | Canaima Nat. Park, Venezuela | P | 46 | 47 | 0.07 |
| [15] | Yakushima Island, Japan | F | 8 | 15 | 0.31 |
| [16] | Brownfield, Illinois, USA | P | 33 | 7 | 0.28 |
| [17] | Santa Genebra Reserve T1. SE Brazil | F | 18 | 7 | 0.30 |
| [17] | Santa Genebra Reserve T2. SE Brazil | F | 29 | 35 | 0.14 |
| [18] | Tropical rainforest, Trinidad. | F | 14 | 50 | 0.33 |
| [19] | Great Britain | F | 14 | 11 | 0.30 |
| [20] | El Viso del Alcor, Sevilla, SE Spain | F | 10 | 14 | 0.46 |

**References**

1. Arroyo MTK, Primack R, Armesto JJ (1982) Community studies in pollination ecology in the high temperate Andes of central Chile. I. Pollination mechanisms and altitudinal variation. Amer J Bot 69: 82-97.
2. Baird JW (1980) The selection and use of fruit by birds in an eastern forest. Wilson Bull 92: 63-73.
3. Beehler B (1983) Frugivory and polygamy in birds of paradise. Auk 100: 1-12.
4. Jordano P, Vázquez D, Bascompte J (2009) Redes complejas de interacciones planta-animal. In: Medel R, Aizen M, Zamora R, editors. Ecología y evolución de las interacciones planta-animal: conceptos y aplicaciones. Santiago: Editorial Universitaria Santiago, Chile. pp. 17-41.
5. Crome FHJ (1975) The ecology of fruit pigeons in tropical Northern Queensland. Aust Wildlife Res 2: 155-185.
6. Guitián J (1983) Relaciones entre los frutos y los passeriformes en un bosque montano de la cordillera cantábrica occidental. PhD Thesis, Univ. Santiago, Spain.
7. Jordano P (1985) El ciclo anual de los paseriformes frugívoros en el matorral mediterráneo del sur de España: importancia de su invernada y variaciones interanuales. Ardeola 32: 69-94.
8. Kevan PG (1970) High Arctic insect-flower relations: the interrelationships of arthropods and flowers at Lake Hazen, Ellesmere Island, Northwest territories, Canada. Ph.D. thesis, University of Alberta, Edmonton.
9. Lambert F (1989) Fig-eating by birds in a Malaysian lowland rain forest. J Trop Ecol 5: 401-412.
10. Olesen JM, Eskildsen LI, Venkatasamy S (2002) Invasion of pollination networks on oceanic islands: importance of invader complexes and endemic super generalists. Diversity Distrib 8: 181-192.
11. Mosquin T, Martin JEH (1967) Observations on the pollination biology of plants on Melville Island, N.W.T., Canada. Can Field Nat 81: 201-205.
12. Percival M (1974) Floral ecology of a coastal scrub in southeast Jamaica. Biotropica 6: 104-129.
13. Tutin CEG, Ham RM, White LJT, Harrison MJS (1997) The primate community of the Lopé Reserve, Gabon: diets, responses to fruit scarcity, and effects on biomass. Am J Primatol 42: 1-24.
14. Ramirez N (1989) Biología de polinización en una comunidad arbustiva tropical de la alta Guyana Venezolana. Biotropica 21: 319-330.
15. Noma N (1997) Annual fluctuations of sapfruits production and synchronization within and inter species in a warm temperate forest on Yakushima Island, Japan. Tropics 6: 441-449.
16. Schemske DW, Willson MF, Melampy MN, Miller LJ, Verner L, et al. (1978) Flowering ecology of some spring woodland herbs. Ecology 59: 351-366.
17. Galetti M, Pizo MA (1996) Fruit eating birds in a forest fragment in southeastern Brazil. Ararajuba, Revista Brasileira de Ornitologia 4: 71-79.
18. Snow BK, Snow DW (1971) The feeding ecology of tanagers and honeycreepers in Trinidad. Auk 88: 291-322.
19. Snow BK, Snow DW (1988) Birds and Berries: a Study of an Ecological Interaction. London: Poyser. 268 p.
20. Herrera CM (1984) A study of avian frugivores, bird-dispersed plants, and their interaction in mediterranean scrublands. Ecol Monogr 54 1-23.
