# Supplementary material for: Do Food Web Models Reproduce the Structure of Mutualistic Networks?
Source: PLoS One. 2011 Nov 2;6(11):e27280. doi: 10.1371/journal.pone.0027280 (PMC3206955; doi:10.1371/journal.pone.0027280)
Supplement: Table S2 — Effects of basic real network features in NME with different N* and M* calculations. (DOC) [file pone.0027280.s006.doc]

Supporting information for “Do food web models reproduce the structure of mutualistic networks?” by MM Pires, PI Prado, PR Guimarães Jr.

|  | *F* | *df* | *r^2^* | *A* | *P* | *C* | *N** | *M** |
| --- | --- | --- | --- | --- | --- | --- | --- | --- |
| Cascade | 69.28** | 22,2 | 0.85 | − | − | 2.21** | -3.11*** | − |
| Niche | 35.94*** | 21,3 | 0.84 | -0.01** | − | − | -1.79*** | 1.82* |
| MPN | 180.90*** | 22,2 | 0.94 | 0.02*** | − | − | -3.14*** | − |
| BC | 18.16*** | 21,3 | 0.68 | − | − | 3.67*** | -0.99* | 4.18 |
| Cascade | 48.28*** | 22,2 | 0.79 | − | − | − | 0.88** | -6.52*** |
| Niche | 44.16*** | 21,3 | 0.84 | 0.02** | − | -1.88** | 1.35*** | − |
| MPN | 48.69*** | 20,4 | 0.88 | 0.02*** | − | -1.35* | 1.26*** | -1.77* |
| BC | 31.89*** | 22,2 | 0.72 | − | − | 2.53** | − | -8.34*** |

**Table S2** Effects of basic real network features in *NME* with different *N** and *M** calculations**.**

GLM results reporting the F-statistics (*F*), degrees of freedom, (*df*), determination coefficient (*r^2^*) and the partial regression coefficients of each factor: Animal species richness (*A*), plants species richness (*P*), connectance (*C*), relative Nestedness (*N**) and relative modularity (*M**). Traces mean that the factor was not included in the best GLM. The significance of each factor and the model as a whole is represented as follows: * < 0.05; ** < 0.01; *** < 0.001. Relative nestedness and modularity were computed using a null model in which the probability of each cell being occupied is the average of the probabilities of occupancy of its row and column [1]

**References**

1. Bascompte J, Jordano P, Melián CJ, Olesen JM (2003) The nested assembly of plant-animal mutualistic networks. Proc Natl Acad Sci U S A 100: 9383-9387.
